# Supplementary figures and images for: Glucosamine Enhancement of Learning and Memory Functions by Promoting Fibroblast Growth Factor 21 Production
Source: Int J Mol Sci. 2024 Apr 10;25(8):4211. doi: 10.3390/ijms25084211 (PMC11050103; doi:10.3390/ijms25084211)

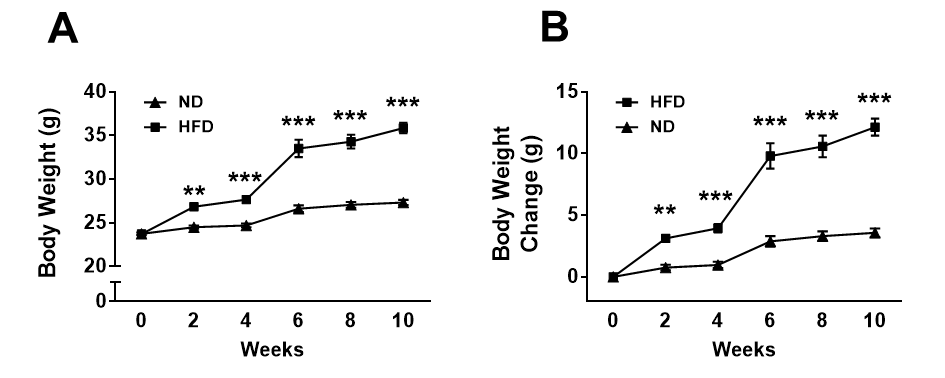

Supplement: Supplementary file 1 [file ijms-25-04211-s001.zip › Supplementary Figure S1.tif]

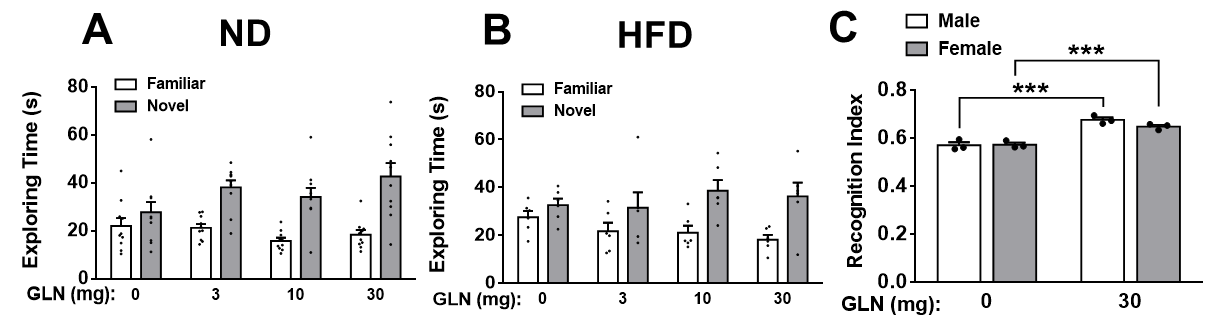

Supplement: Supplementary file 1 [file ijms-25-04211-s001.zip › Supplementary Figure S2.tif]

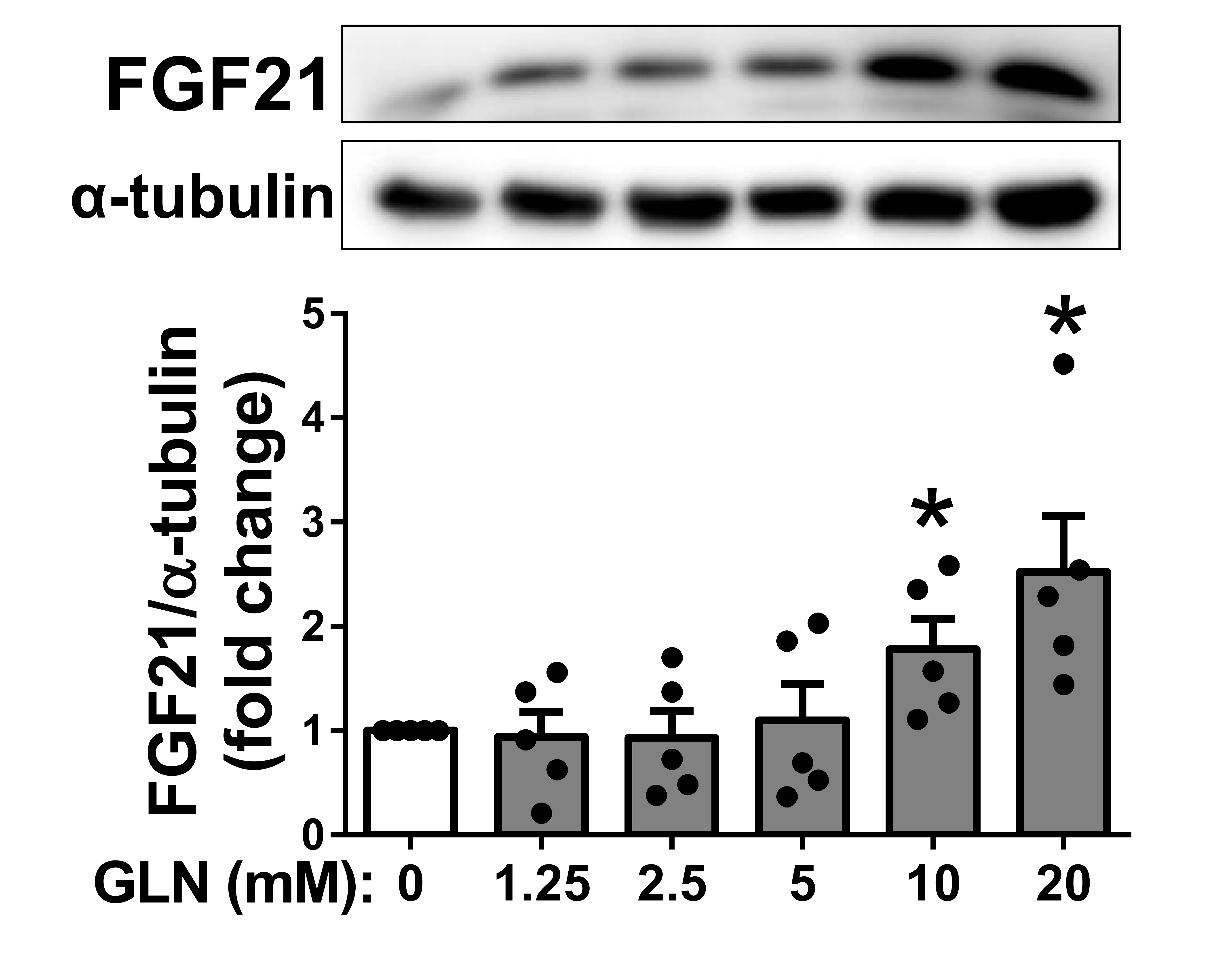

Supplement: Supplementary file 1 [file ijms-25-04211-s001.zip › Supplementary Figure S3.tif]

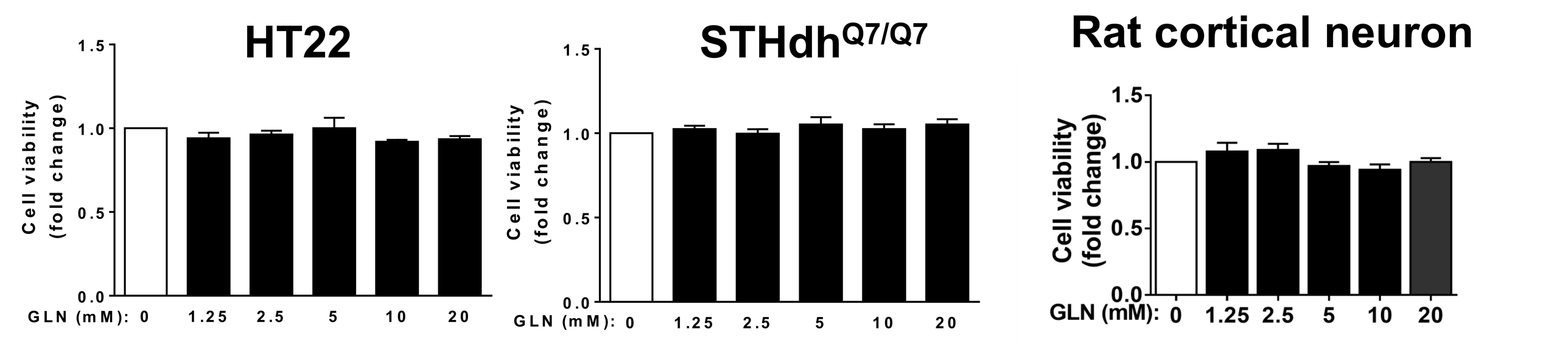

Supplement: Supplementary file 1 [file ijms-25-04211-s001.zip › Supplementary Figure S4.tif]

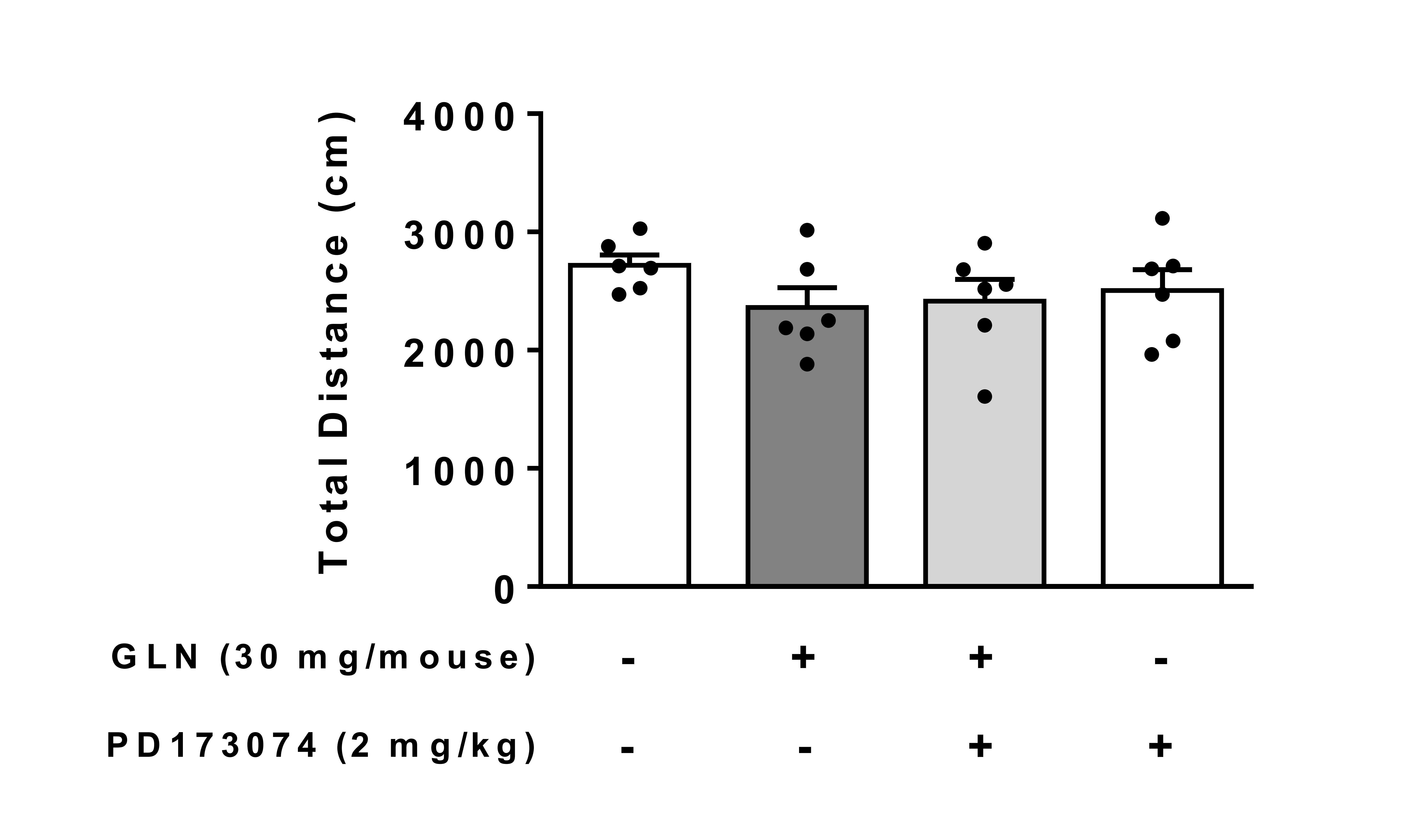

Supplement: Supplementary file 1 [file ijms-25-04211-s001.zip › Supplementary Figure S5.tif]

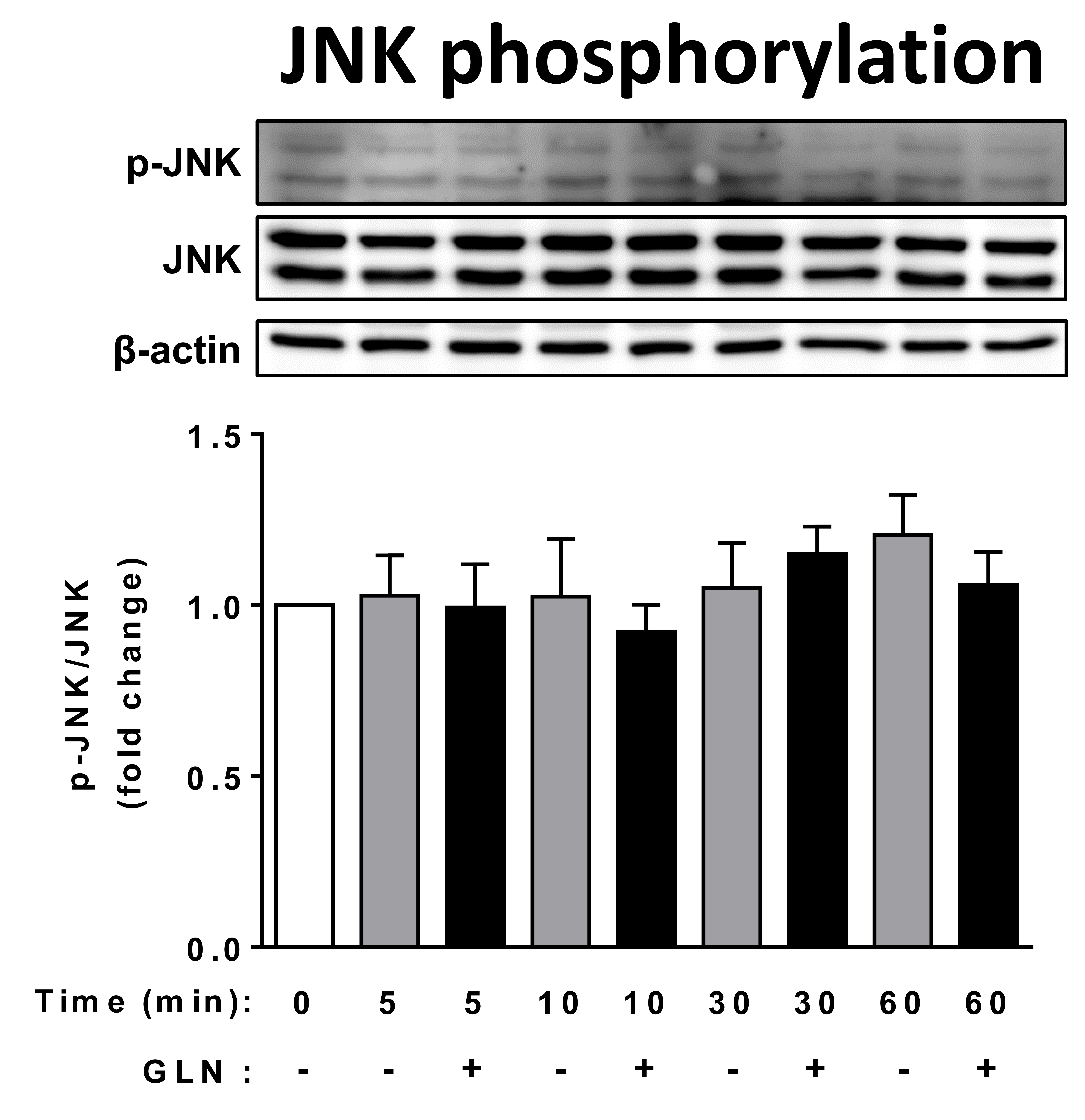

Supplement: Supplementary file 1 [file ijms-25-04211-s001.zip › Supplementary Figure S6.tif]

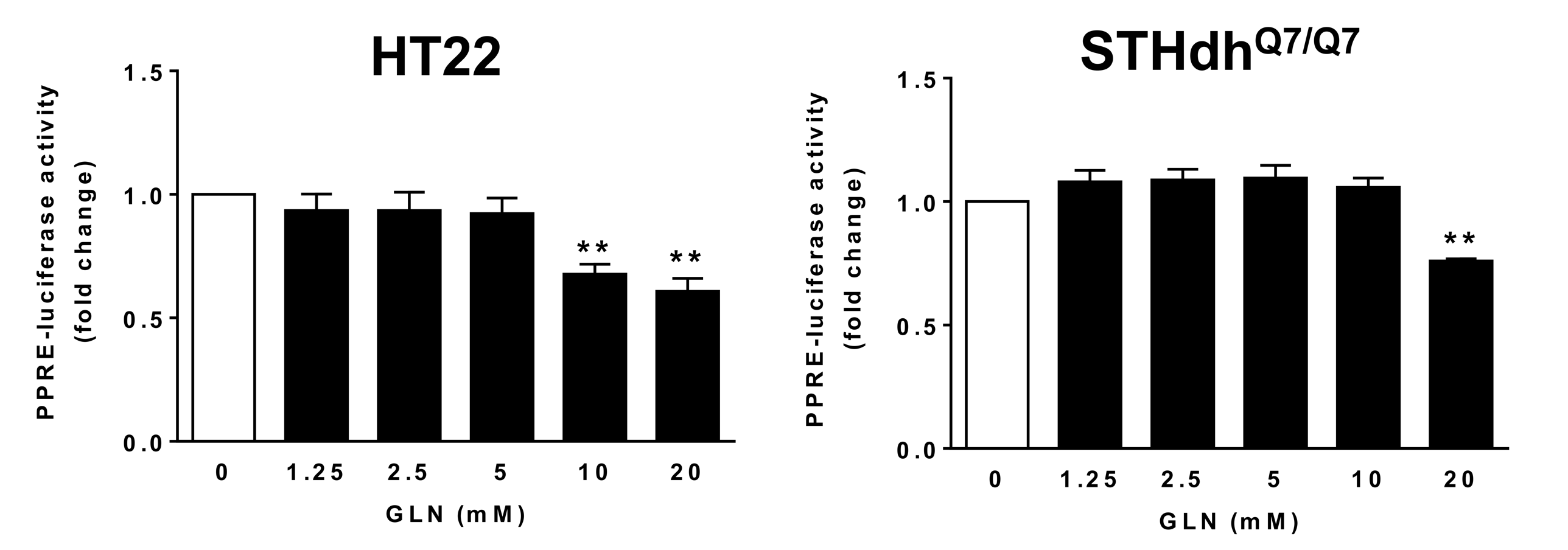

Supplement: Supplementary file 1 [file ijms-25-04211-s001.zip › Supplementary Figure S8.tif]
